# Quantitative dynamic near-infrared fluorescence imaging using indocyanine green for analysis of bowel perfusion after mesenteric resection

**DOI:** 10.1117/1.JBO.26.6.060501

**Published:** 2021-06-09

**Authors:** Ruben P. J. Meijer, Labrinus van Manen, Henk H. Hartgrink, Jacobus Burggraaf, Sylvain Gioux, Alexander L. Vahrmeijer, J. Sven D. Mieog

**Affiliations:** a Leiden University Medical Center, Department of Surgery, Leiden, The Netherlands; b Centre for Human Drug Research, Leiden, The Netherlands; c University of Strasbourg, ICube Laboratory, Strasbourg, France

**Keywords:** bowel perfusion, near infrared fluorescence, indocyanine green, mesenteric resection

## Abstract

**Significance:** Near-infrared (NIR) fluorescence imaging using indocyanine green (ICG) has proven to be a feasible application for real-time intraoperative assessment of tissue perfusion, although quantification of NIR fluorescence signals is pivotal for standardized assessment of tissue perfusion.

**Aim:** Four patients are described with possible compromised bowel perfusion after mesenteric resection. Based on these patients we want to emphasize the difficulties in the quantification of NIR fluorescence imaging for perfusion analysis.

**Approach:** During image-guided fluorescence assessment, 5 mg of ICG (2.5  mg/ml) was intravenously administered by the anesthesiologist. NIR fluorescence imaging was done with the open camera system of Quest Medical Imaging. Fluorescence data taken from the regions of interest (bowel at risk, transition zone of bowel at risk and adjacent normally perfused bowel, and normally perfused reference bowel) were quantitatively analyzed after surgery for fluorescence intensity-and perfusion time-related parameters.

**Results:** Bowel perfusion, as assessed clinically by independent surgeons based on NIR fluorescence imaging, resulted in different treatment strategies, three with excellent clinical outcome, but one with a perfusion related complication. Post-surgery quantitative analysis of fluorescence dynamics showed different patterns in the affected bowel segment compared to the unaffected reference segments for the four patients.

**Conclusions:** Similar intraoperative fluorescence results could lead to different surgical treatment strategies, which demonstrated the difficulties in interpretation of uncorrected fluorescence signals. Real-time quantification and standardization of NIR fluorescence perfusion imaging could probably aid surgeons in the nearby future.

## 1 Introduction

Extensive oncological abdominal surgery occasionally includes removal of part of the mesentery containing blood vessels supplying the associated bowel. Because mesenteric resection can result in compromised bowel perfusion leading to necrosis, it is commonly combined with bowel resection and subsequent anastomosis formation. However, anastomosis formation harbors the risk of an anastomotic leakage, which is the most feared complication of bowel resections and accounts for considerable morbidity and mortality.^1^^–^^3^ Indirect perfusion of adjacent bowel segments with intact mesenterial blood supply could be sufficient to avoid bowel resection and subsequent anastomosis after partial mesenteric resection. For decades, surgeons rely on the use of tactile and visual feedback to determine the perfusion state, which has certain limitations. Near-infrared (NIR) fluorescence imaging using indocyanine green (ICG) has proven to be a feasible and reproducible application for real-time intraoperative quantification of tissue perfusion.^4^^–^^6^ Surgical procedures in which part of the mesentery is resected can benefit from NIR fluorescence imaging with ICG. However, quantification of NIR fluorescence signals is pivotal for standardized assessment of tissue perfusion. This report aims to demonstrate the current shortcomings in intraoperative quantification of NIR fluorescence imaging for bowel perfusion assessment after mesenterial disruption without a planned bowel resection.

## 2 Methods and Case Reports

### 2.1 Methods

Four patients underwent standard-of-care NIR fluorescence imaging using ICG for intraoperative bowel perfusion assessment after mesenteric resection. Informed consent for publication of this work was obtained from the patients. For the surgical procedures, 25 mg of ICG (Pulsion Medical Systems, Munich, Germany) was diluted in 10 ml sterile water to obtain a 2.5  mg/ml concentration. During image-guided fluorescence assessment, 5 mg of ICG was intravenously administered by the anesthesiologist. NIR fluorescence imaging was done with the open-camera system of Quest Medical Imaging (Middenmeer, The Netherlands).

Fluorescence data were quantitatively analyzed after surgery for fluorescence intensity-and perfusion time-related parameters according to Son et al.^7^ Snapshots were taken every 2 s after ICG injection from the video recording, and fluorescence intensities of each image were sequentially analyzed using Mevislab (MeVis Medical Solutions AG, Bremen, Germany). Five regions of interest were taken: one from the part of the bowel at risk, two from the margin of adjacent normally perfused bowel to bowel at risk (the transition zone), and two from normally perfused bowel adjacent to the bowel at risk. Results were averaged in case of two regions per zone. The following fluorescence parameters were analyzed: Half of difference between baseline and maximum fluorescence intensity ($F_{1/2\;\max}$), difference between baseline and maximum fluorescence intensity ($F_{\max}$), time from first fluorescence increase to maximum intensity ($T_{\max}$), slope of the fluorescence inflow ($\text{slope}=F_{\max}/T_{\max}$), slope in 10 s after the rise of fluorescence intensity (${\text{slope}}_{10}$), time from first fluorescence increase to half of maximum intensity ($T_{1/2\;\max}$), and the ratio of $T_{1/2\;\max}$ over $T_{\max}$ ($\mathrm{TR}=T_{1/2\;\max}/T_{\max}$).

### 2.2 Case I

**Fig. 1:**
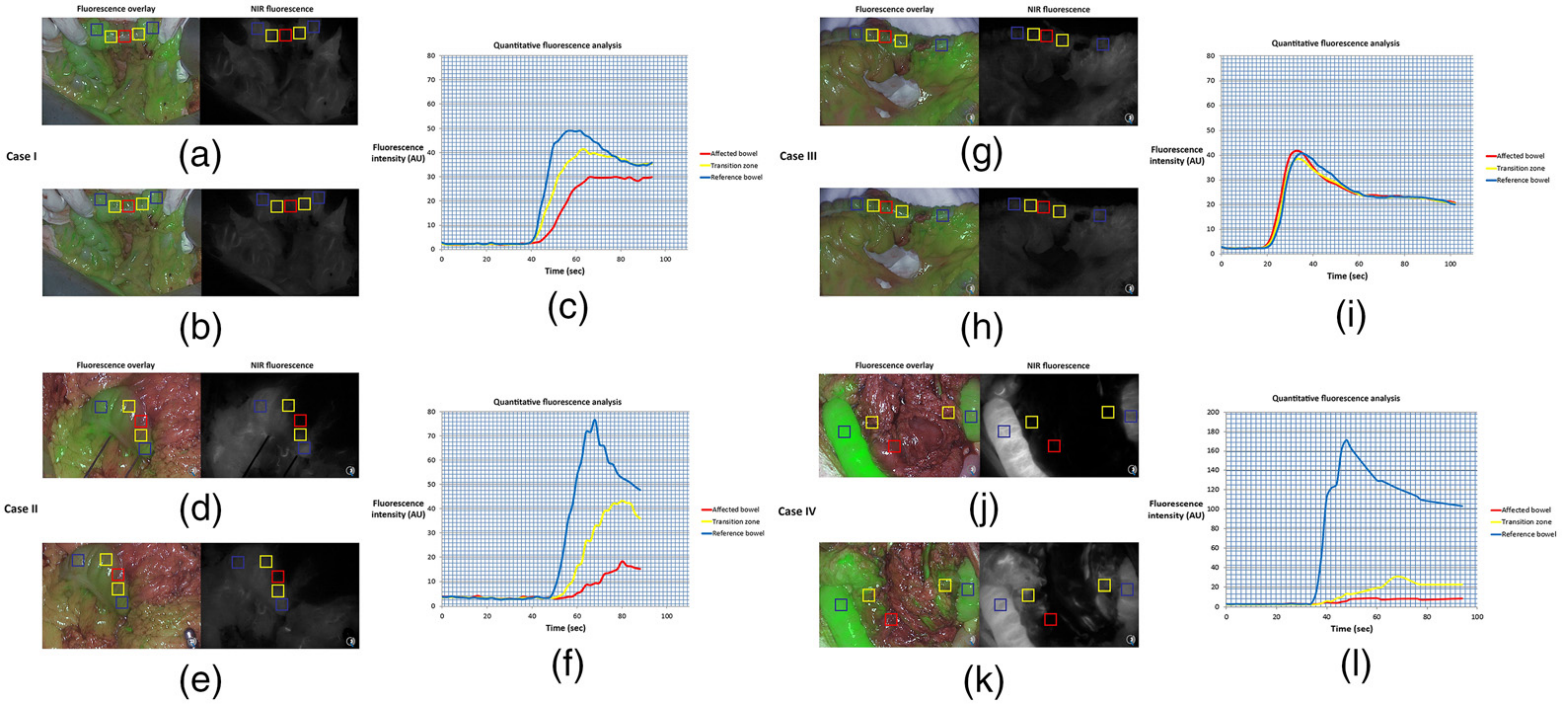
Overview of ICG inflow and quantitative perfusion analyses. (a), (d), and (j) Case I, II, and IV showed a delayed fluorescence inflow; (b), (e), and (k) lower maximum fluorescence intensity levels in the affected bowel, which is illustrated by (c), (f), and (l) quantitative analysis. Case III showed minimal difference in (g) fluorescence inflow or (h) maximum fluorescence intensity. ROIs were taken in the affected bowel with compromised perfusion (red), transition zone with possible impaired perfusion (yellow), and reference bowel with normal perfusion (blue). Abbreviations: ICG: indocyanine green; ROI: region of interest; AU: arbitrary units.

A 25-year-old man, who underwent several resections for recurrent osteosarcoma, was referred for resection of a progressively growing abdominal nodule of 21 mm in size detected on routine computed tomography that was suspected to be a metastatic lesion. At explorative midline laparotomy, no other metastases were found. The lesion was situated in the mesocolon adjacent to the descending colon and consequently a mesenteric resection sparing the left colic artery was performed. Visual assessment of the colon showed slight discoloration at the resection side over a trajectory of 4 cm. Intraoperative subjective fluorescence assessment of the bowel perfusion showed slightly slower increase of fluorescence intensity of the bowel segment at risk (Fig. 1), but this part became as fluorescent as the reference bowel within one minute. The perfusion was judged as being sufficient and it was decided to not perform a bowel segment resection. The postoperative period was uneventful, and discharge was at postoperative day 3.

Postoperative quantitative fluorescence analysis showed lower maximum fluorescence intensities (27.3 versus 39.2 versus 50.8 AU), inflow slope (0.76 versus 1.56 versus 2.56  AU/s), and ${\text{slope}}_{10}$ (1.05 versus 2.04 versus 3.74  AU/s) in the affected bowel compared to the transition zone and reference bowel (Table 1). Furthermore, $T_{\max}$ (36 versus 25 versus 19 s), $T_{1/2\;\max}$ (12 versus 9.5 versus 7.5 s) and TR (0.33 versus 0.38 versus 0.39) were lower at the transition zone and directly perfused reference bowel segments.

**Table 1:** Quantitative perfusion analysis of described cases compared to literature for anastomotic leakage.

		Case I			Case II			Case III			Case IV			Literature comparison
		Affected bowel	Transition zone	Reference bowel	Affected bowel	Transition zone	Reference bowel	Affected bowel	Transition zone	Reference bowel	Affected bowel	Transition zone	Reference bowel	Suggested cutoff values for poor perfusion
Fluorescence intensity related factors														
	$F_{1/2\;\max}$ (AU)	13.7	19.5	24.2	7.4	20.2	36.5	19.5	18.1	19.2	2.75	16.5	84.9	NA
	$F_{\max}$ (AU)	27.3	39.2	50.8	14.8	40.4	73.0	38.9	36.2	38.3	5.5	32.9	169.8	<52^8^
	Slope or ingress rate (AU/s)	0.76	1.56	2.56	0.57	1.35	3.65	2.43	2.15	2.30	0.23	0.97	12.13	<0.7^7^; <2.1^8^
	${\text{Slope}}_{10}$ (AU/s)	1.05	2.04	3.74	0.51	1.28	3.82	3.11	2.67	2.42	0.21	0.97	12.02	NA
Perfusion time-related factors														
	$T_{\max}$ (s)	36	25	19	26	30	20	16	17	17	24	35	14	>57^8^
	$T_{1/2\;\max}$ (s)	12	9.5	7.5	17	15	10	8	8.5	10	12	14.5	7.5	>18^7^; >14^8^; >10^9^
	TR	0.33	0.38	0.39	0.65	0.50	0.50	0.50	0.50	0.59	0.5	0.43	0.54	>0.6^7^

Abbreviations: $F_{1/2\;\max}$: half of difference between baseline and maximum fluorescence intensity; NA: not available; $F_{\max}$: difference between baseline and maximum fluorescence intensity; AU: arbitrary units; slope: slope of the fluorescence inflow; ${\text{slope}}_{10}$: slope in 10 s after the rise of fluorescence intensity, $T_{\max}$ : time from first fluorescence increase to maximum intensity; $T_{1/2\;\max}$: time from first fluorescence increase to half of maximum intensity; TR: ratio of $T_{1/2\;\max}$ and $T_{\max}$.

### 2.3 Case II

A 71-year-old man, without any relevant other diagnosis in his medical history, was referred because of esophageal cancer, eligible for resection after neoadjuvant treatment. During the dissection of the greater gastric curvature, iatrogenic injury of the mesocolon occurred. Visual assessment of the colon showed an area of about 3 cm that was slightly darker than the surrounding bowel. Intraoperative fluorescence assessment of the bowel perfusion showed decreased fluorescence intensity adjacent to the aperture in the mesocolon (Fig. 1, lower panel). It was concluded that perfusion of this part of the bowel was inadequate and therefore the two well-perfused parts were approximated with serosal sutures, thereby folding the less perfused part intraluminally. The postoperative recovery was uneventful, and discharge was at postoperative day 7.

Postoperative quantitative fluorescence analysis showed a difference in $F_{\max}$ (14.8 versus 40.4 versus 73.0 AU), slope (0.57 versus 1.35 versus 3.65  AU/s), and ${\text{slope}}_{10}$ (0.51 versus 1.27 versus 3.82  AU/s) between the bowel segment at risk, the transition zone, and the unaffected bowel segment (Table 1). $T_{\max}$ was higher at the transition zone compared to the affected bowel and reference bowel (30 versus 26 versus 20 s). $T_{1/2\;\max}$ was higher for the affected bowel compared to the transition zone and reference bowel (17 versus 15 versus 10 s). TR was equal between the transition zone and reference bowel (0.50), although TR was higher (0.65) for the affected bowel segment.

### 2.4 Case III

A 53-year-old female patient underwent a pylorus-preserving pancreaticoduodenectomy because of an adenocarcinoma of the duodenum. A partial resection of the mesentery of the transverse colon including the right colonic artery was unavoidable due to tumor involvement. Visually, no problems were suspected regarding the perfusion of the adjacent bowel segment. Assessment of the perfusion using ICG-guided fluorescence showed clear and equal fluorescence in all parts of the bowel (affected and unaffected zones). As a result, no further intraoperative intervention was deemed necessary. During her recovery the patient did not develop any symptoms of inadequate bowel perfusion.

Postoperative quantitative fluorescence analysis showed similar maximum fluorescence intensities (38.9 versus 36.15 versus. 38.3 AU), inflow slope (2.43 versus 2.15 versus 2.30  AU/s), and ${\text{Slope}}_{10}$ (3.11 versus 2.67 versus 2.42  AU/s) in the affected bowel compared to the transition zone and reference bowel (Table 1). Furthermore, $T_{\max}$ (16 versus 17 versus 17 s), $T_{1/2\;\max}$ (8 versus 8.5 versus 10 s) and TR (0.5 versus 0.5 versus 0.59) did not show any clinical significant difference at three different zones of the bowel.

### 2.5 Case IV

A 76-year-old male patient, diagnosed with gastric cancer, underwent residual stomach resection, because of a tumor-positive resection margin after previous distal gastrectomy 3 months before. During the current surgical procedure part of the mesentery of the transverse colon had to be resected, due to extensive adhesions, as the patient had multiple abdominal surgeries in the past. At visual inspection the adjacent transverse colon was darker than the assumed healthy bowel segments and arterial pulsations were weak. Subjective intraoperative fluorescence assessment of the bowel perfusion showed delayed fluorescence inflow of a 3-cm long transverse colon segment. Subjective fluorescence intensity seemed equal at all bowel sites 3 min after ICG administration. It was decided not to intervene, based on doubtful palpable pulsations and fluorescence signals after 3 min. Two days postoperative a relaparotomy was performed, which showed an ischemic part of the transverse colon of 3 cm and poor perfusion over 10 cm, which was resected and subsequently an anastomosis was created. The patient recovered and was discharged 2 weeks after the initial surgery.

Postoperative quantitative fluorescence analysis showed decreased maximum fluorescence intensities (8.8 versus 36.1 versus. 173.25 AU), inflow slope (0.23 versus 0.97 versus 12.13  AU/s), and ${\text{Slope}}_{10}$ (0.21 versus 0.97 versus 12.02  AU/s) in the affected bowel and transition zone compared to reference bowel (Table 1). $T_{\max}$ (24 versus 35 versus 14 s), $T_{1/2\;\max}$ (12 versus 14.5 versus 7.5 s) and TR (0.5 versus 0.43 versus 0.54) did not show any clinical significant difference at three different zones of the bowel.

## 3 Discussion

In this report, four patients with clinically suspect compromised bowel perfusion after mesenteric resection are described. Bowel perfusion, as assessed by independent surgeons based on NIR fluorescence imaging, resulted in different treatment strategies, which are supported for case I, II, and III by quantitative fluorescence analysis as compared to current literature. The affected bowel in patient I and II showed slightly impaired perfusion parameters, which was more pronounced in the second patient. The third patient did not show any impaired perfusion parameters and might therefore be considered as a negative control in patients with normal bowel perfusion after mesenteric resection. Patient IV showed distinct poor perfusion parameters. Unfortunately, conventional methods, including interpretation of the unquantified NIR fluorescence imaging withheld the surgeon from doing a resection with subsequent anastomosis. Patient IV also emphasizes an important drawback of subjective NIR fluorescence assessment. After time, ICG tends to distribute even to ischemic zones which possibly leads to overestimation of the perfusion quality at the end of ICG distribution.

Quantification of NIR fluorescence signals is pivotal for standardized assessment of tissue perfusion. Currently, available fluorescence imaging systems lack the capacity to perform real-time quantitative imaging during surgery. Therefore, surgeons have to assess the fluorescence signal in a qualitative manner using the perceived intensity and inflow as the only parameters. This can easily lead to misinterpretation as fluorescence intensities vary greatly with the experimental conditions and do not take into account the time-dependent diffusion of ICG. More precisely, fluorescent intensity is dependent on type and amount of injected tracer, the camera-target distance, the local tissue absorption, and scattering properties and camera system specific settings including, but not limited to the excitation light delivery mode and fluorescence light collection.^10^^–^^13^

Quantitative fluorescence parameters are categorized in time-dependent and fluorescence intensity-related that have already been investigated during reconstructive, vascular, and intestinal surgery in several studies.^7^^,^^10^^,^^13^^–^^15^ For bowel surgery, fluorescence parameters have mainly been correlated to the occurrence of anastomotic leakage and have to our knowledge not yet been described in patients with mesenteric resection, in which collateral vessels provide perfusion to the affected bowel segment. It has been suggested that a smaller fluorescence inflow slope (arterial inflow) and a low $F_{\max}$ are important predictors for poor vascular perfusion.^8^^,^^10^ Three studies described cutoff values for these fluorescence parameters to determine higher risk of either colorectal or free jejunal graft anastomotic leakage, even though these studies used different cutoff values and demonstrated contradictory results.^7^^–^^9^ Slope (<0.7  AU/s; <2.1  AU/s)^7^^,^^8^ and $T_{1/2\;\max}$ (>18  s; >10  s)^7^^,^^9^ were significantly associated with anastomotic leakage in two out of three studies, whereas $F_{\max}$ (<52  AU)^8^ and TR (>0.6)^7^ were significantly associated in one out of three studies. Although these cutoff values are not directly applicable to our cases, it provides a general indication of bowel perfusion.

According to the flow chart proposed by Son et al.,^7^ the affected bowel segment in the second case could be described as a zone with an acceptable perfusion, to which surgeons should pay extra attention. In the current cases, the surgeons decided on different treatment strategies after subjective fluorescence assessment. Post-surgical analysis showed mildly compromised tissue perfusion of the affected bowel segments in patient I and II, but more pronounced in the second case. One could say that the surgeons interpreted the fluorescence images sufficiently, as the surgeon of the second case decided for a small intervention. Moreover, good clinical outcome in case I indicates sufficient indirect perfusion of adjacent bowel segments, justifying the decision not to intervene.

As the area of the resected mesenteric layers was quite small in the four cases (∼5×5  cm), it would be worthwhile to further investigate the effect of the size of the resected mesentery on the perfusion fluorescence parameters at certain distances on the bowel and correlate this to serum lactate levels in more controlled circumstances.^10^^,^^13^ Moreover, patient-related factors, such as smoking or hypertension,^16^ could also influence the bowel perfusion and should also be considered when analyzing the bowel perfusion using NIR fluorescence imaging.

Current quantification methods include temporal analysis of the fluorescence dynamic signal,^13^ such as in this study, ratiometric imaging using an additional reflectance channel to normalize the fluorescence signal,^17^ and quantitative fluorescence imaging using spectral, or spatial modulation of light.^12^ It is important to acknowledge both benefits and disadvantages of each quantification method. The analysis of the fluorescence dynamics in time allows to quantify the temporal properties of the contrast agent diffusion within the tissues. Though this is a powerful method to assess adequacy of perfusion, the advantage may be off-set as it necessitates point measurements precluding real-time availability of results or a complex acquisition and processing methodology to gather all the necessary information and display results as a quantitative heatmap. Ratiometric imaging has the advantage of being simple to implement and allows to eliminate variation factors such as distance or illumination inhomogeneity, but it has to be acknowledged that tissue optical properties (absorption and scattering) are not fully addressed.^17^

Soon, improvements in imaging devices and quantitative imaging methods will provide more reliable and interpretable information content in real-time during surgery. Both quantitative fluorescence and dynamic fluorescence imaging are demonstrating the potential of optical imaging to augment the capacity for surgeons to observe objectively and identify problems during the surgery. Systems able to display the dynamic evolution of the fluorescent signal, such as fluorescence-based enhanced reality (FLER) provide a quantitative and reproducible estimation of the bowel perfusion in augmented reality.^18^ FLER is independent from the distance, since it is time-dependent and independent from the camera-to-target distance. Nevertheless, improvement in quantification methods are necessary to move the field forward. It is suggested that correcting for arterial input function by pulse dye densitometry could improve the currently used quantification methods by reducing the intra- and inter-subject variability.^19^ Furthermore, fluorescence imaging can be combined with other methods such as endogenous functional imaging of oxygenation or blood perfusion to increase the capabilities of optical imaging to guide surgery reliably and in real time.^20^^,^^21^

## 4 Conclusion

This report demonstrates the difficulties of visual assessment of fluorescence perfusion imaging in patients with potentially compromised bowel segment following partial mesenteric excision. In the presented cases, a bowel resection with subsequent anastomosis was not planned, which makes it difficult to compare our results with existing literature. Moreover, the currently available quantitative fluorescence parameters are sparsely studied, and their cutoffs differ significantly among studies warranting validation in large clinical trials. More fundamental studies are needed on underlying kinetic mechanisms causing the variance in these parameters.
